# Seroprevalence of Toxocariasis and Its Associated Risk Factors among Adult Population in Kavar District, Fars Province, South of Iran: A Cross-Sectional Community-Based Seroepidemiological Survey

**DOI:** 10.1155/2023/2721202

**Published:** 2023-05-10

**Authors:** Fatemehsadat Pezeshkian, Ali Pouryousef, Mostafa Omidian, Fattaneh Mikaeili, Ali Reza Safarpour, Sara Shojaei-Zarghani, Bahador Sarkari

**Affiliations:** ^1^Student Research Committee, Shiraz University of Medical Sciences, Shiraz, Iran; ^2^Department of Parasitology and Mycology, School of Medicine, Shiraz University of Medical Sciences, Shiraz, Iran; ^3^Gastroenterohepatology Research Center, Shiraz University of Medical Sciences, Shiraz, Iran; ^4^Colorectal Research Center, Shiraz University of Medical Sciences, Shiraz, Iran; ^5^Basic Sciences in Infectious Diseases Research Center, Shiraz University of Medical Sciences, Shiraz, Iran

## Abstract

Toxocariasis as a common neglected disease is the culprit of infecting all age groups. The current cross-sectional study was designed to assess the seroprevalence of toxocariasis and risk factors associated with seropositivity of *Toxocara* infection among the general population of adults in the Kavar district, south of Iran. A total of 1060 participants with an age range of 35 to 70 years from the Kavar region entered the study. Manual ELISA was utilized to detect anti-*Toxocara*-specific antibodies in their serum samples. In addition, demographic information as well as risk factors related to toxocariasis was collected from individuals who participated in the survey. The mean age of the participants was 48.9 (±7.9) years old. Out of 1060 subjects, 532 (50.2%) were men, and 528 (49.8%) were women. The overall *Toxocara* seroprevalence was 5.8% (61/1060). The prevalence of *Toxocara* seropositive cases was significantly different between males and females (*p* = 0.023). The seropositive rate for *Toxocara* infection was also significantly higher in housewives (*p* = 0.003) and subjects with learning disabilities (*p* = 0.008). Multivariable logistic regression revealed that housewives (OR = 2.04, 95% CI: 1.18–3.51, *p* = 0.010) and subjects with learning disability (OR = 3.32, 95% CI: 1.29–8.52, *p* = 0.013) were at increased risk of *Toxocara* infection. The findings of the current study depicted a noticeable seroprevalence of *Toxocara* infection in the general population in the Kavar district, southern Iran. An increased risk of toxocariasis has been associated with learning disabilities and being a housewife. All of the toxocariasis-positive cases had contact with animals, at some point in their life. In perspective, it is necessary to raise awareness of this infection among the population while surveilling *Toxocara* infection in high-risk groups.

## 1. Introduction

*Toxocara canis and T. cati* are common parasites of dogs and cats with global prevalence while its prevalence in both animals and humans is highest in developing countries [1]. These intestinal nematodes are transmitted to humans through the ingestion of embryonated eggs nested in contaminated raw vegetables or soil [2, 3]. Moreover, geophagia and poor personal hygiene have also been observed to contribute to the attainment of this parasitic infection. Furthermore, humans could also become infected by ingesting undercooked or raw meat infected with L3-stage larvae as well as organs from paratenic hosts (e.g., rabbits, pigs, cattle, and chickens) [4]. Later on, once the embryonated eggs are ingested, the larvae hatch and migrate through the somatic organs. The clinical manifestations of human toxocariasis are dependent on the infected organ (liver, eye, brain, and other human organs) [5]. Visceral larva migrans (VLM) and ocular larva migrans (OLM) are the two major complications of this infection. However, most cases encounter toxocariasis asymptomatically as covert toxocariasis [5, 6].

Toxocariasis is a neglected, yet significant tropical infection with a global prevalence. A high distribution of toxocariasis is observed in both industrial and developing countries [7]. The seroepidemiology of toxocariasis in different geographic regions is diverse, which has been reported from 3% to 79% globally with an upstream in Asian countries [8]. In a study, 22.3% of suspected people in Bulgaria were *Toxocara* seropositive [9]. Thus far, numerous studies have been conducted on the seroepidemiology of toxocariasis in Iran. The reported seroprevalence of human toxocariasis in Iran is 9.3% with a range from 0.84 to 29% [10, 11]. The seroprevalence of *Toxocara* infection in pet owners in western Iran was reported to be 20.43%, while the contamination prevalence in the control group, with no contact with dogs or cats, was 1.07% [12]. The findings of various studies depict Iran as one of the noteworthy *Toxocara* endemic countries in the Middle East [13]. However, there is a call for further population studies on the seroprevalence of *Toxocara* infection in Iran, to obtain a comprehensive map of the prevalence of human toxocariasis in different areas of the country, especially small towns and remote villages.

Studies conducted in Fars province, one of Iran's largest and most populous provinces, illustrate a noteworthy rate of toxocariasis among the definitive hosts, humans, and also in the soil of the region [14–18]. Toxocariasis prevalence in school children aged 6 to 13 years in Shiraz, the capital of Fars province, was reported to be 25.6% [16]. Our previous study observed a 26.3% *Toxocara* seropositivity in municipal street sweepers in the same area [17]. It has also been reported that 26.7% of stray cats in Shiraz were infected with *T. cati* [14].

Kavar, a small, remote district in Fars province, is a symbol of animal husbandry and agricultural lifestyle with its old texture where stray dogs and cats roam everywhere in groups with no control measure. This pattern of lifestyle increases the risk of parasitic zoonotic infections such as toxocariasis, cystic echinococcosis, and leishmaniasis. Conclusively, the lack of information about the prevalence of *Toxocara* infection in the above-mentioned district led us to investigate the seroprevalence and risk factors of human toxocariasis, in the form of a population-based study, among the general population of this remote area in Fars province, south of Iran.

## 2. Materials and Methods

### 2.1. Study Area and Sample Collection

To identify eligible participants for the current cross-sectional study, the necessary coordination was made with researchers of the Kavar Persian population cohort. Two main research centers, the Gastroenterohepatology and Endocrine Research Centers linked to Shiraz University of Medical Sciences (SUMS), are in charge of the comprehensive Persian cohort study.

Kavar's population is about 71,856, and the district is located 35 kilometers southeast of the capital of Fars province, Shiraz. The districts geographical coordinates are latitude 11°29′N and longitude 42°52′E. The district has a large number of stray cats and dogs that are in close contact with humans and other animals, including sheep and goats. The source of income for the people living in Kavar is agriculture, animal husbandry, and cattle breeding.

Between 2017 and 2019, in the baseline phase of the mentioned cohort study, 4,997 individuals aged 35–70 residing in Kavar city were enrolled. To minimize selection bias and increase representativeness, all residents of the city were informed regarding the study, and all eligible subjects were recruited (census). From this group, and without any exclusions, 1,060 cases were randomly selected using SPSS software. The sample size was calculated using the following formula:

(1) $$\begin{array}{c} n=\frac{Z^{2}\;p\left(1-p\right)}{d^{2}}\text{.} \end{array}$$

**Figure 1:**
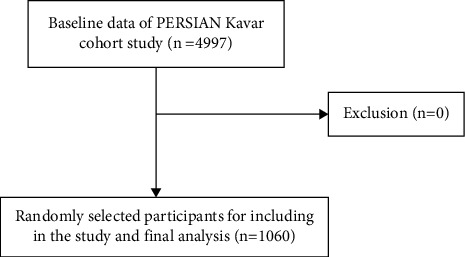
Flow diagram of the study participants.

Given the reported prevalence of *Toxocara* infection in Iran (9.3%) [13], 95% confidence interval (CI), and precision (*d*) of 0.02, the sample size was calculated to be 810. With consideration of a dropout rate of 30%, 1060 subjects were included in the study. A blood sample of 5 mL was taken from each participant after obtaining ethical consent to assess anti-*Toxocara* antibodies (as shown in Figure 1). While considering the cold chain, the sera samples were kept at −20°C until the test was carried out, after being transferred to the laboratory of the Department of Parasitology and Mycology, at Shiraz Medical School. Demographic characteristics of the participants along with risk factors for toxocariasis such as age, gender, occupation, medical history, animal contact history, and even the type of contact were extracted from the cohort database [19]. The animal contact options provided in the questionnaire were classified as follows: (1) sometimes (i.e., animals residing within 200 meters of home or workplace); (2) not daily, but at least twice each month (i.e., animals residing next to home or workplace); (3) daily (i.e., animals kept in the house or workplace, but participant is not involved in taking care of the animal); (4) close daily contact (i.e., feeding or cleaning animal living area).

Ethical approval of the study was obtained from the Ethical Review Committee of Shiraz University of Medical Sciences (Ethics Committee Registration Code: IR.SUMS.MED.REC.1401.397).

### 2.2. Preparation of Antigen and Detection of AntiToxocara Antibodies

First, the second-stage larvae of *Toxocara *excretory-secretory antigens (TES) were prepared based on the method of Zibaei et al. [20]. Briefly, the uteri of female worms were dissected, and the isolated eggs were placed in a 2.5% formalin ringer and then incubated at 25°C for 30 days. Larvae were recovered by Baermann apparatus and were cultivated in Roswell Park Memorial Institute (RPMI) 1640 medium. After collecting the supernatant culture containing TES antigens, purification and storage at −20°C were performed. Indirect ELISA, using flat-bottom 96-well microplates coated with 5 *μ*g/mL of the *Toxocara* ES antigens in coating buffer (0.05 M carbonate-bicarbonate buffer, pH 9.6), was developed for detecting anti-*Toxocara* antibodies in serum samples. Sera were kept at 4°C for 24 hours. The following day, using the washing solution (PBST: 0.05% Tween 20 in PBS) and with the help of an ELISA washer, the plates were washed three times automatically. Blocking was performed using 5% skimmed milk. After incubation at room temperature (RT) for 2 h, the plates were washed as before, and then, diluted serum samples (1/100 dilution in PBST buffer) were added to the plates as well. Meanwhile, *Toxocara* positive and negative controls were used. After washing as explained previously, anti-human IgG-horseradish peroxidase conjugate (Sigma, USA; 1/4000 in PBST) was added and incubated in RT for one hour. In the next step, substrate solution containing OPD/H_2_O_2_ (100 *μ*L/well of 0.4 mg/mL OPD, 0.3% H_2_O_2_ in 0.1 M citrate buffer, pH 5) was added, and then, the plate was placed in darkness in RT for 15 min. The absorbance was read at 490 nm using a microplate reader (ELX800, BioTek, USA). Eventually, the cut-off value was determined by the mean of negative controls OD value plus two standard deviations (SDs).

### 2.3. Statistical Analysis

The normality of the distribution of continuous variables was evaluated by descriptive statistics. Qualitative and parametric data are expressed as numbers (percentages) and mean ± SD. The Chi-square and Fisher's exact tests were employed for categorical data. Univariate and multivariable logistic regression models (backward) were carried out to disclose the association between toxocariasis and potential risk factors (*p* values for entry and removal set to 0.05 and 0.10, respectively). Values are expressed as odds ratio (OR) and 95% CI. IBM SPSS (version 26.0) was used for data analysis. A two-sided *p* value <0.05 was considered significant.

## 3. Results

The study population was 1060 participants, including 532 (50.2%) males and 528 (49.8%) females living in Kavar County, Fars province, southern Iran. The mean age of the participants was 48.9 (±7.9) years, ranging between 35 and 70 years old. Most of the cases (43.8%) belonged to the age group of 46–55 years. People were divided into eight categories according to their job, which is illustrated in Table 1. Accordingly, most of the subjects (45.8%) were in the housewife group. Anti-*Toxocara* antibodies were detected in the sera of 61 out of 1060 samples, corresponding to a seroprevalence rate of 5.8%. Seropositivity to toxocariasis was more prevalent in the age group 35–45 (7%) but was statistically insignificant. The seropositivity rate of toxocariasis in females (7.4%) was statistically higher than the males' subjects (4.1%) (*p* < 0.05).

The seroprevalence of toxocariasis was higher in subjects with learning disabilities (18.8%) than in those without learning disabilities (5.4%), and the difference was statistically significant (*P* value = 0.008). Seropositivity to *Toxocara* in subjects with and without chronic lung disease was 12.1% and 5.6%, respectively, but the difference was statistically insignificant (*P* value = 0.116). Among the population with rheumatic disease, anti-*Toxocara* antibodies were more prevalent than those without rheumatic disease, 9.8% versus 5.6%. However, the difference was not statistically significant (*P* value = 0.209). Toxocariasis seropositivity was slightly higher amongst subjects who had psychiatric disorders, depression, and amnesia than those without mental disorders, but it was statistically insignificant. The further statistical report is depicted in Table 2.

The results of our study indicated that female sex (*P* = 0.025), learning disability (*P* = 0.003), and housewifery (*P* = 0.004) were significantly positively associated with seropositivity of toxocariasis. Furthermore, through multivariable logistic regression analysis, we have found that learning disability (OR = 3.32, 95% CI: 1.29–8.52, *P* = 0.013) and housewifery (OR = 2.04, 95% CI: 1.18–3.51, *P* = 0.010) remained independently associated with toxocariasis (Table 3) (age and sex did not remain in the model).

## 4. Discussion

Toxocariasis, as one of five important cosmopolitan neglected diseases by the Centers for Disease Control and Prevention (CDC), poses a considerable threat to public health [21]. The seroprevalence of human toxocariasis in different regions can be diverse owing to differences in factors such as soil contamination rate, temperature, humidity range, and level of social hygiene. However, toxocariasis could occur globally, in any age group, due to the high infection rate in definitive hosts and also the presence of different paratenic hosts, which is a source of concern.

Only one-third of the studies have been dedicated to detecting *Toxocara* antibodies in the adult population in Iran [22]. This is the first population-based study conducted to investigate the seroprevalence of toxocariasis in a large number of adults by taking underlying diseases into account in the Kavar area, a remote and somewhat nomadic area. On the other hand, adults usually present with the covert form of toxocariasis, which makes toxocariasis underdiagnosed in these groups compared to children. As predicted, *Toxocara* prevalence in our studied population was relatively high (5.8%) in comparison with those studies which reported the rate of *Toxocara* seroprevalence in 5–15 years old children in Isfahan (1.39%), in rural inhabitants of Khuzestan Province, southwest Iran (2%), in children (3–13 years old) in Zahedan, southeast of Iran (1.3%), in children under 14 years referring to laboratories of Sistan and Baluchestan Province in southeast of Iran (1.7%), and among nomads in Boyer-Ahmad County, southwest Iran (1.4%) [23–27]. However, the seroprevalence rate of *Toxocara* in our study is far lower than the prevalence reported in some parts of Iran, including in children (5–15-year-old) in Ardabil district, northwest of Iran (14%), in children from urban and rural areas of Ilam Province, west Iran (22%), or among mentally retarded patients in Hormozgan Province, Southern Iran (28.2%) [28–30]. The results of a study in northeastern Iran revealed that living in rural areas is one of the risk factors for toxocariasis [31]. In Vietnam, the proportion of toxocariasis seropositivity was in favor of countryside inhabitants with a prevalence of 79.26%, compared to 20.76% in those who lived in urban areas [32]. Our previous study on municipality workers in Fars province, in the south of Iran, revealed a high prevalence of toxocariasis, 26.3%, in participants [17].

Based on our findings, age was not a significant risk factor for toxocariasis. However, a population-based study in northeastern Iran indicated younger age as a significant risk factor for toxocariasis [31]. Also, in other parts of the world, some studies have shown that seroprevalence of toxocariasis tended to increase with age [33, 34]. In line with this report, a study in Brazil revealed a significant increase in toxocariasis prevalence in the adult population (17.4%) compared to children (4.2%) [35].

The findings of the current study have shown that the female gender was one of the factors significantly associated with the prevalence of toxocariasis in the univariate, but not multivariable, analysis. A study in Vietnam displayed that 62.24% of females tested positive for toxocariasis, as opposed to 37.76% of male society [32]. An acceptable justification for this association is that most women, in our study area, due to being housewives are exposed to contaminated vegetables regardless of personal hygiene measures and tend to gravitate toward taking care of pets and animals in their homes or farms [32]. In this regard, housewives were at two-times increased risk of *Toxocara* infection in our study.

In our study, all individuals who tested positive for toxocariasis reported that they sometimes had a contact with dogs or cats. In the region where the present study was conducted, stray dogs with a large population are roaming freely in all areas. Furthermore, the presence of cats in the courtyards and around the houses is an inseparable part of life in these areas.

Animals, particularly cats and dogs, are the major source of infection for the *Toxocara* life cycle and are considered an eligible risk factor for toxocariasis and other soil-transmitted parasites [31, 36]. Inspecting toxocariasis in cat owners in the capital of Iran, Tehran, disclosed that 51% of the owners were infected with toxocariasis [37]. Furthermore, a significant association has been reported between *Toxocara* seropositivity and owning a dog [38]. Assessing the awareness of pet owners regarding toxocariasis revealed a disturbing lack of awareness which contributes to increased *Toxocara* infection [39].

Perhaps, one of the important findings of the present study is that it revealed a significant association between learning disability and *Toxocara* infection where individuals with learning disabilities had the highest rate of *Toxocara* seropositivity rate in comparison with those patients who had other underlying diseases. People with learning disabilities are usually those with intellectual disabilities. These people are mostly maintained in institutions with unfavorable living conditions which increase their chances of getting different infectious diseases including *Toxocara* infection. Behavioral factors, such as nail-biting and sucking fingers, along with inadequate personal hygiene in these people lead to an increasing risk of *Toxocara* infection in this high-risk group. In our previous study that was performed on 117 mentally retarded patients, 28.2% of them were infected with toxocariasis [29].

It is important to note that all subjects enrolled in our study had similar prior history of exposure to animals. Therefore, based on our findings, we cannot determine a definitive association between different levels of exposure to animals and *Toxocara* infection. Additionally, the current study has a limitation in terms of age groups as subjects between the age groups of 18–34 years and above 70 years were not included. Therefore, the interpretation of results should be conducted with caution. Another limitation of this study is the relatively long period of sample preparation, which is due to the requirements of the designed cohort. To comprehend a seroprevalence map for toxocariasis, further longitudinal studies taking into account the rural areas in various locations are required.

## 5. Conclusion

Our study found that the general population of Kavar County has an overall *Toxocara* seroprevalence of 5.8%. In addition, we observed a positive association between *Toxocara* infection prevalence and both learning disability and housewifery. According to the findings of the study, it can be concluded that in addition to children, adults also are at a noteworthy risk for toxocariasis. Therefore, it seems imperative to discover groups at risk on a larger scale and improve their education and improving lifestyle. A distinct focus should be bestowed on rural area inhabitants' education and awareness along with screening and monitoring toxocariasis in the animals that are in contact with humans.

## Figures and Tables

**Table 1:** Demographic features of the total included subjects and those with toxocariasis.

Characteristics		Frequency (percent %)	Positive for anti-Toxocaraantibodies (percent %)	*P* value^*∗*^
Age (years)	35–45	344 (32.5)	24 (7.0)	0.470
	46–55	464 (43.8)	23 (5.0)	
	≥56	252 (23.8)	14 (5.6)	

Sex	Male	532 (50.2)	22 (4.1)	**0.023**
	Female	528 (49.8)	39 (7.4)	

Job	Housewife	486 (45.8)	39 (8.0)	**0.003**
	Retired	170 (16.0)	11 (6.5)	0.662
	Driver	94 (8.9)	2 (2.1)	0.160
	Employee	69 (6.5)	3 (4.3)	0.792
	Freelance occupation	95 (9.0)	5 (5.3)	0.829
	Labor	63 (5.9)	0 (0.0)	**0.045**
	Farmer	72 (6.8)	1 (1.4)	0.117
	Stockman	11 (1.0)	0 (0.0)	1.000

Animal contact with cats and dogs (sometimes)		1060 (100)	61 (5.0)	

^*∗*^ *P* value <0.05 was considered significant. The comparison was conducted between subjects with positive and negative anti-Toxocara antibodies. Bold values represent p < 0.05, which was considered statistically significant.

**Table 2:** Prevalence of underlying diseases in the total included subjects and those with toxocariasis.

Underlying diseases	Frequency (percent %)	Positive for anti-*Toxocara*antibodies (percent %)	*P* value^*∗*^
Learning disabilities	32 (3.0)	6 (18.8)	**0.008**
Psychiatric disorders	74 (7.0)	6 (8.1)	0.432
Amnesia	101 (9.5)	9 (8.9)	0.173
Rheumatic disease	51 (4.8)	5 (9.8)	0.209
Chronic lung disease	33 (3.1)	4 (12.1)	0.116
Depression	167 (15.8)	12 (7.2)	0.368
Epilepsy	16 (1.5)	1 (6.3)	0.615
Chronic headache	135 (12.7)	8 (5.9)	0.845
Cancer	6 (0.6)	0 (0.0)	1.000
Fatty liver	184 (17.4)	12 (6.5)	0.603
Renal disease	15 (1.4)	2 (13.3)	0.212
Cardiac disease	77 (7.3)	3 (3.9)	0.616
Hypertension	198 (18.7)	10 (5.1)	0.737
Diabetes	168 (15.8)	11 (6.5)	0.591

^*∗*^ *P* value <0.05 was considered significant. Comparison was conducted between subjects with positive and negative anti-Toxocara antibodies. Bold values represent p < 0.05, which was considered statistically significant.

**Table 3:** Univariate and multivariable logistic regression for risk factors of toxocariasis.

Variables	Univariate analysis		Multivariable analysis	
	OR (95% CI)	*P* value	OR (95% CI)	*P* value
Age (continues)	0.99 (0.96–1.02)	0.547		
Sex (female vs. male)	1.85 (1.08–3.16)	0.025		
Learning disabilities (yes vs. no)	4.08 (1.61–10.33)	0.003	3.32 (1.29–8.52)	**0.013**
Job (housewives *vs.* others)	2.19 (1.28–3.75)	0.004	2.04 (1.18–3.51)	**0.010**

CI: confidence interval; OR: odds ratio. *P* value <0.05 was considered significant. Bold values represent p < 0.05, which was considered statistically significant.

## Data Availability

The data supporting the current study are available from the corresponding author upon request.

## Supplementary Materials

Supplementary Materials STROBE Statement—Checklist of items that should be included in reports of cross-sectional studies.
